# Over-Expression of Centromere Protein U Participates in the Malignant Neoplastic Progression of Breast Cancer

**DOI:** 10.3389/fonc.2021.615427

**Published:** 2021-03-23

**Authors:** Xiaomeng Hao, Yufan Qiu, Lixia Cao, Xiaonan Yang, Dongdong Zhou, Jingjing Liu, Zhendong Shi, Shaorong Zhao, Jin Zhang

**Affiliations:** ^1^ Third Department of Breast Surgery, Tianjin Medical University Cancer Institute and Hospital, National Clinical Research Center for Cancer, Tianjin, China; ^2^ Key Laboratory of Cancer Prevention and Therapy, Tianjin, China; ^3^ Tianjin’s Clinical Research Center for Cancer, Tianjin, China; ^4^ Key Laboratory of Breast Cancer Prevention and Therapy, Tianjin Medical University, Ministry of Education, Tianjin, China

**Keywords:** CENP-U, proto-oncogene, breast cancer, progression, PI3K/AKT/NF-κB pathway

## Abstract

The expression of Centromere Protein U (CENP-U) is closely related to tumor malignancy. Till now, the role of CENP-U in the malignant progression of breast cancer remains unclear. In this study, we found that CENP-U protein was highly expressed in the primary invasive breast cancer tissues compared to the paired adjacent histologically normal tissues and ductal carcinoma *in situ* (DCIS) tissues. After CENP-U was knocked down, the proliferation and colony-forming abilities of breast cancer cells were significantly suppressed, whereas the portion of apoptotic cells was increased. Meanwhile, the PI3K/AKT/NF-κB pathway was significantly inhibited. *In vivo* studies showed that, the inhibition of CENP-U repressed the tumor growth in orthotopic breast cancer models. Therefore, our study demonstrated that the CENP-U might act as an oncogene and promote breast cancer progression *via* activation of the PI3K/AKT/NF-κB pathway, which suggests a promising direction for targeting therapy in breast cancer.

## Introduction

Centromeres, the essential chromosomal domains in cell mitosis and meiosis, provide a structural platform for kinetochore assembling to guarantee the correct chromosome segregation (1, 2). The centromeres are DNA/protein complexes where a class of highly conserved protein called centromere-associated proteins (CENPs) is deposited (3, 4). The kinetochore, the CENPs assembled at each centromere along with multiple other kinds of proteins that are involved in the microtubule attachment. CENPs can act as the attachment site for spindle microtubules and also can serve as the site at which produces force to drive chromosome movement during mitosis (5–7). In addition to effects on mitosis, CENPs was shown to play an important part in tumorigenesis and progression in a large volume of literature. Centromere protein A (CENP-A) is over-expressed in colorectal cancer cell lines and suggested to be an independent prognostic marker for patients with estrogen receptor (ER)-positive breast cancer who received no systemic therapy (8, 9). Many previous studies have reported a high expression level of centromere protein E (CENP-E) and centromere protein W (CENP-W) in several solid tumors such as uterine cervical cancer, gastric cancer, and hypopharyngeal cancer (10–16). Furthermore, centromere protein H (CENP-H) has been demonstrated to be a novel biomarker related to the survival in patients with hepatocellular carcinoma (17).

Centromere protein U (CENP-U), an important resident centromere-binding protein, is one of the important elements composing constitutively centromere-associated network (CCAN) in kinetochore lining, which participates in assemble of the middle mature kinetochore, takes an important role in cellular mitosis and regulates cell-cycle status by recruiting function-specific proteins. Recent studies indicated the expression of CENP-U went together with malignancy. In Banin Hirata BK et al.’s research (18), microarray analysis was performed using Affymetrix U133A microarray in 200 breast cancer patients, and found that *CENP-U* gene was significant over-expressed in recurrent patients compared with no-recurrent ones, indicating that CENP-U could be a prognostic and predictive marker for breast cancer patients. Chou et al. (19) aggregated microarray datasets from the Gene Expression Omnibus and selected 21 most-associated genes to predict breast cancer recurrence including CENP-U gene. Previous studies have shown that CENP-U is over-expressed in breast cancer (20, 21) and associated with the survival of patients (22–25) as well as in lung cancer (26, 27).

On the basis of previous studies, the purpose of this research is to demonstrate the effects and underlying molecular mechanism of CENP-U in breast cancer cells proliferation *in vivo* and *in vitro*, which would be beneficial to the recognition of CENP-U functions for discovering a new target for the breast cancer research in initiation and development.

## Materials and Methods

### Sample Tissue

Sixty samples of primary breast cancer tissues (30 cases of paraffin-embedded invasive ductal breast carcinoma (IDC) and 30 cases of ductal carcinoma *in situ* (DCIS) tissues and the paired adjacent histologically normal tissues were achieved from Tianjin Medical University Cancer Institute and Hospital between January 2008 and January 2010, and no presurgical chemotherapy, radiotherapy, or endocrine therapy were applied on the patients. This study has been approved by the Institutional Review Board of Tianjin Medical University Cancer Institute and Hospital, and has obtained written consents from all participants.

### Cell Lines and Animals

The human breast cancer cell lines T47D, MDA-MB-231, SK-BR-3, MCF-7, the corresponding multi-drug resistant (MDR) counterpart MCF-7/ADR, and human mammary epithelial cell line MCF-10A were obtained from American-Type Culture Collection (Manassas, VA, USA). The breast cancer cell line CAL51 and its MDR counterpart CALDOX were generously provided by Dr. Ernesto Yague (Imperial College London, London, UK). HEK293T was achieved from the Type Culture Collection of the Chinese Academy of Sciences (Shanghai, China). All of the cells were cultured as previously described before (28, 29) at 37°C in a humidified atmosphere of 5% CO_2_.

The female BALB/c nude mice of 4–6 weeks old with body weight of 20 ± 2 g were bought from the Department of Laboratory Animal Science, Peking University Health Science Centre [license number: SCXK (Beijing) 2006–2008]. The animal experiments were performed in compliance with the animal care and use committee of Tianjin Medical University Cancer Institute and Hospital.

### Plasmids, shRNA, Lentivirus Production, and Transduction

For the over-expression of CENP-U, the coding sequence of human CENP-U (Biolight, Nanjing, China) was cloned into the lentiviral expression vector pCDH-CMV-MCS-EF1-Puro (System Biosciences, CA, USA). According to the manufacturer’ instructions, a mixture of CMV-dR8.91, pCMV-VSV-G, and pCDH-CENP-U or pCDH-CMV-MCS-EF1-Puro was transfected into HEK293T cells using Lipofectamine 2000 (Invitrogen) to produce lentivirus. T47D and MCF-10A cells were infected with recombinant lentivirus-transducing plus 8 mg/ml polybrene (Sigma). For the knockdown of CENP-U, shRNA plasmid, shControl plasmid, and lentiviral packaging system were bought from Genechem (Shanghai, China). According to the Genechem’s manufacturer’s instructions, the packaged lentiviruses were obtained after shCENP-U/shControl cotransfection with lenti-Easy Packaging Mix for 48 h to infect MDA-MB-231 and MCF-7 cells.

### Immunohistochemistry

Immunohistochemistry staining was done in the method of streptavidin-peroxidase (SP) using CENP-U antibody (YN1585, 1:250, Immunoway) for all specimens. Instead of the CENP-U antibody, phosphate buffered saline (PBS) was used as a negative control. CENP-U is located in both cytoplasm and nucleus (30–32). The criteria for assessing positive cells were the presence of brown or dark brown yellow particles in the cytoplasm or nucleus. Five microscopic fields were randomly examined at 400×, and the percentage of positive-stained area was counted. The quantity score ranges from 0 to 4: 0, no immunostaining; 1, 1–14% of the areas are positive; 2, 15–49% of the areas are positive; 3, 50–74% of the areas are positive; and 4, ≥75% of the areas are positive.

### Cell Proliferation Assays

For observe and compare cell proliferation ability, both growth curve and colony formation assays were implemented. Cells were seeded onto 24-well plates at the density of 5 × 10^3^ cells/well and the number of survival cells was counted daily for five days after trypan blue staining.

Cells were seeded onto six-well plates at the density of 500 cells/well. One week later, cells were fixed by methanol for 10 min and stained by 0.005% crystal violet for 20 min. The colony number was counted at 100× microscope field, as the cell number of per colon should be more than 30.

### MCF-10A Colony Formation in Soft Agar

Basic agar was prepared by mixing a volume of 1:4 of molten 3% agar and medium. Then 2 ml basic agar was added in each well of six-well plates at 37°C for 1 h. The final concentration of top Agar in the DMEM-agar mixture was 0.3%, and 1 × 10^4^ MCF-10A cells were seeded in the top agar. The colonies were counted 5 weeks later (21).

### Flow Cytometry Analysis

For cell cycle analysis, the cells were fixed with cold 70% ethanol for 24 h and added in a 1 ml solution containing 1 mg/ml propidium iodide (PI), 50 μg/ml RNase and PBS. To detect apoptosis, the suspension of freshly prepared viable cells in 500 μl Binding buffer including 5 μl Annexin V-FITC and 5 μl PI was conducted. Cell cycle and apoptosis were analyzed by fluorescence-activated cell sorter (FACS).

### Western Blotting Analysis

Cells and tissues were lysed in RIPA buffer (Solarbio, Beijing, China), and proteins were separated by SDS-PAGE and transferred to polyvinyldi-fluoride membranes (Millipore, USA), and then the membranes were blocked with 5% blotting-grade milk for 1 h. the membranes were incubated overnight at 4°C with rabbit antibodies against CENPU (1:2,000, YN1585, Immunoway), rabbit antibodies against PI3K-p110α (1:1,000, YT3709, Immunoway), rabbit antibodies against AKT1 (1:1,000, YT0177, Immunoway), rabbit antibodies against pAKT Ser473 (1:1,000, YP0006, Immunoway), mouse antibodies against S6 (1:1,000, 2317S, Cell Signaling Technology), rabbit antibodies against pS6 Ser235/236 (1:1,000, YP0243, Immunoway), rabbit antibodies against NF-κB p65 (1:1,000, 8242S, Cell Signaling Technology), mouse antibodies against β-actin (1:1,000, 3700S, Cell Signaling Technology). The blots were then washed and incubated for 1 h at room temperature with peroxidase-conjugated anti-rabbit IgG and anti-mouse IgG secondary antibody (Servicebio, GB23301 and GB23303, respectively, 1:3,000). The concentration of protein fragments was measured using Image-Pro Plus Ver. 7.0 software. URL: https://www.mediacy.com/imageproplus.

### Immunofluorescence Staining

MCF-10A cells were trypsinized and plated onto six-well plates. After the cells were cultured until reaching 50% confluence, we fixed them with 2% paraformaldehyde for 15 min and permeabilized with 0.2% Triton X-100 in PBS for 5 min at room temperature (33). Then samples were blocked with 3% bovine serum albumin (BSA) in PBS for 30 min (34). To detect the CENP-U, samples were incubated with an anti-CENP-U primary antibody for 1 h at room temperature, followed by incubation with Alexafluor488-labeled secondary antibody (Santa Cruz Biotechnology, Dallas, USA) at room temperature for 1 h. Besides, the nuclei were stained with 1 mg/ml 4’,6-Diamidino-2-phenylindole (DAPI) (Sigma-Aldrich) for 10 min. All the stains and antibodies we have used were diluted in 3% BSA/PBS, and the coverslips were coated with ProLong Gold Anti-Fade Reagent. Between the above staining steps, the sample were washed three times with PBS. Finally, immunofluorescent images were observed using a Zeiss LSM 510 Metamicroscope (Zeiss, Jena, Germany).

### Xenograft Tumor Assay

To assess the effect of CENP-U on breast cancer growth *in vivo*, 231-shCENP-U/231-shControl or T47D-CENP-U/T47D-Control cells suspension of 5 × 10^6^ cells in 100 μl (including 50% Matrigel) was injected hypodermically into the left mammary fat pads of BALB/c nude mice. One week after injected, the weight of mice, the maximum diameter (Length), and the minimum diameter (width) of the xenograft tumor were meteraged every 3 days for 18 days. The tumor volume (mm^3^) was counted on the basis of the formula: volume (mm^3^) = 1/2 × length × width2.

### Statistical Analysis

Analyses were performed using the Statistical Product and Service Solutions Ver. 18.0 software (IBM Inc., Armonk, NY, USA). URL: https://www.ibm.com/cn-zh/analytics/spss-statistics-software. Statistical significance between two groups and multiple groups were analyzed by Student’s t test and ONE-WAY-ANOVA. All probability values in this study were bidirectional. Besides, all the experiments were carried out for three times and the data were in the form of mean ± standard deviation (SD). The p-value <0.05 as threshold for statistical significance.

## Results

### CENP-U Protein Is Expressed Differently in Adjacent Normal Tissue, DICS, and IDC

**Figure 1:**
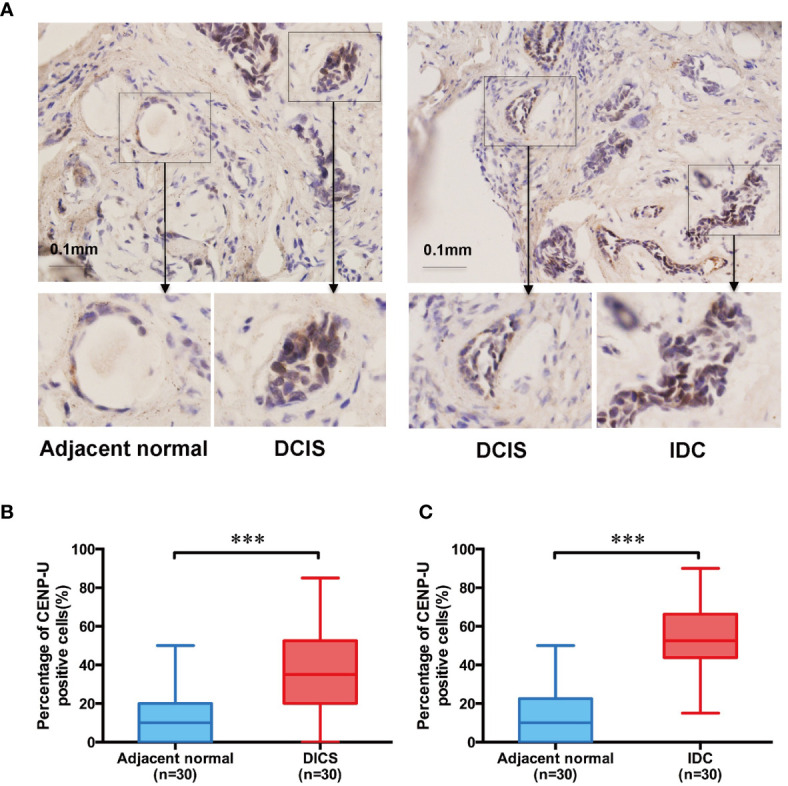
The CENP-U protein expression in DICS, IDC, and paired adjacent histologically normal tissues. **(A)** Expression of CENP-U in DICS, IDC, and paired adjacent histologically normal tissues were detected using immunohistochemical staining. Nuclei were counterstained with hematoxylin (blue) (magnification: ×200). **(B)** The percentage of CENP-U positive cells in DICS and the paired adjacent normal tissues (n = 30) were measured by IHC. **(C)** The percentage of CENP-U positive cells in IDC and the paired adjacent normal tissues (n = 30) were measured by IHC. The statistical analysis was performed with Student’s *t*-test. NS, not significant; ***P < 0.001 compared with the adjacent normal tissue group.

To explore the impact of CENP-U in breast cancer tumorigenesis, we used immunohistochemistry to determine CENP-U protein expression in both cancer tissues and the paired adjacent histologically normal tissues of 30 patients with IDC and 30 with DCIS (**Figure 1A**). The percentage of CENP-U positive cells in both DICS and IDC tissues were higher than the paired adjacent histologically normal tissues (*P* < 0.001) (**Figure 1B**) respectively. Intriguingly, a notable increased CENP-U expression was observed in IDC compared to DICS (*P* < 0.001) (**Figure 1C**). CENP-U expression in cancer cells was significantly higher than in normal tissues and the number of CENP-U positive cells in IDC were also higher than DICS, suggesting that CENP-U expression level was up-regulated with the development of breast cancer.

### CENP-U Is Over-Expressed in Breast Cancer Cell Lines

**Figure 2:**
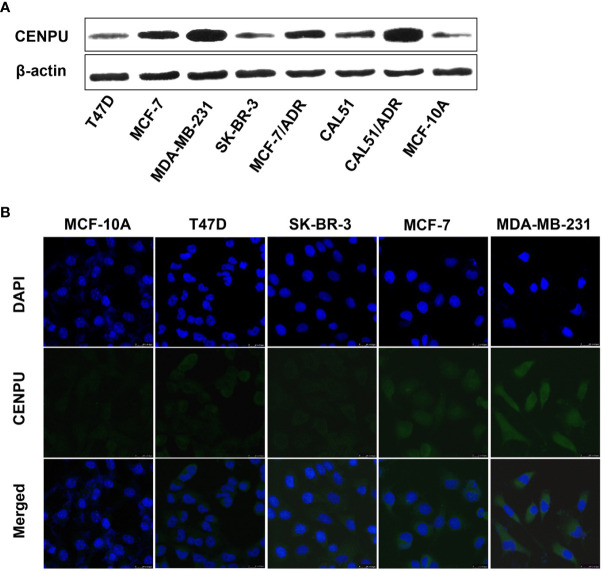
The expression and location of CENP-U protein in normal epithelial cell line and breast cancer cell lines. **(A)** CENP-U protein expression in breast cancer cell lines was detected by western blotting. **(B)** CENP-U protein (green) was located immunocytochemically in both the nucleus and cytoplasm of breast cancer cells and normal epithelial cells. Nuclei were counterstained with DAPI (blue) (magnification: ×400).

To investigate the relationship between CENP-U expression status and tumorigenesis of breast cancer, we detected the different expression levels of CENP-U in breast cancer cell lines and normal epithelial cell line MCF-10A by Western blotting (**Figure 2A**). Compared with breast cancer cell lines, the expression of the CENP-U protein decreased significantly in normal breast epithelial cell line MCF-10A. In breast cancer cell lines, the level of CENP-U expression was elevated in MCF-7, MDA-MB-231, and CAL51 and decreased in T47D and SK-BR-3. We also compared the expression of CENP-U between chemo-sensitive cell lines (MCF-7 and CAL51) as well as MDR breast cancer cell lines (MCF-7/ADR and CALDOX). A significantly increased CENP-U expression was examined in MDR triple negative breast cancer cell lines compared with chemo-sensitive cell lines. The location of CENP-U protein in breast cancer cells were investigated by immunofluorescence staining. The results showed that CENP-U was not only present in the nucleus, but also highly expressed in the cytoplasm of four breast cancer cell lines and normal epithelial cell line (**Figure 2B**). The immunofluorescent analysis also showed that the expression of CENP-U was highest in MDA-MB-231 cell line.

These data indicated that the expression of CENP-U was lowest in the MCF-10A cell line and was highest in the MDA-MB-231 cell line.

### Depletion of CENP-U Expression Inhibits Breast Cancer Cell Proliferation, Results in Cell Cycle Arrest in G2/M Phase, and Induces Apoptosis In Vitro

**Figure 3:**
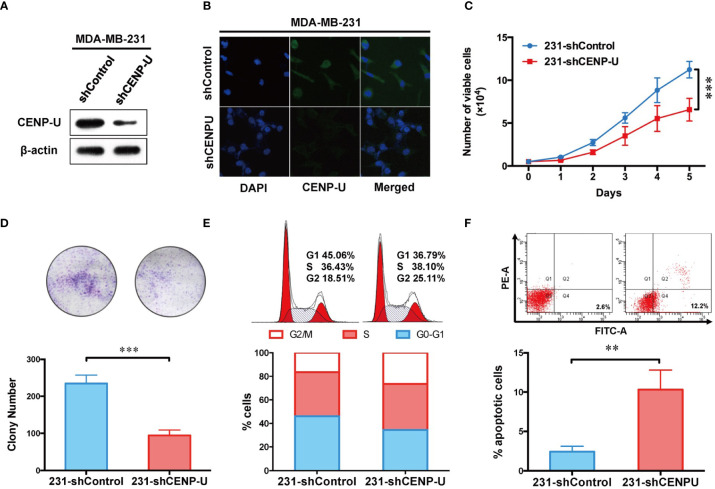
Depletion of CENP-U expression inhibits breast cancer cell proliferation, leads to cell cycle arrest in G2/M phases and induces early apoptosis *in vitro*. **(A)** CENP-U knockdown was confirmed by western blotting in stably transfected MDA-MB-231 cells. **(B)** Down-regulation of CENP-U expression did not change the location of CENP-U protein in MDA-MB-231 cells by immunofluorescence. **(C)** The cell growth curve of 231-shControl cells and 231-shCENP-U cells. ***P = 0.0005. **(D)** The colony number of 231-shControl cells and 231-shCENP-U cells. **(E)** Cell cycle of 231-shControl cells and 231-shCENP-U cells analysis were detected by flow cytometry. **(F)** Apoptosis analysis of 231-shControl cells and 231-shCENP-U cells were performed by flow cytometry. The statistical analysis was performed with Student’s t-test. **P < 0.01, ***P < 0.001.

To explore the impact of CENP-U in tumorigenesis of breast cancer cells, we used CENP-U siRNA plasmids to establish stable CENP-U depletion cells. Previous data indicated that CENP-U was highly expressed in the MDA-MB-231 and MCF-7 cells (35, 36). Therefore, we established stable CENP-U depletion in MDA-MB-231, MCF-7 cells (231-shCENP-U/MCF-7-shCENP-U) and the negative control cells (231-shControl/MCF-7-shCcontrol). CENP-U knockdown was confirmed by western blot in the subclones of MDA-MB-231 (**Figure 3A**) and MCF-7 (**Figure S1A**). Immunofluorescence analysis confirmed that down-regulation of CENP-U expression did not change the location of CENP-U protein (**Figure 3B** and **Figure S1B**). Then, we analyzed the role of CENP-U knockdown on breast cancer cell proliferation and colony formation. The cell growth curve indicated that 231-shCENP-U cells grew significantly more slowly than 231-shControl cells (*P* = 0.0005) (**Figure 3C**), and MCF-7-shCENP-U and MCF-7-Control cells also showed the same trend (*P* = 0.0162) (**Figure S1C**). Colony formation was found by culturing shCENP-U cells and shControl cells for 2 weeks. Compared with the 231-shControl cells, the colony number of 231-shCENP-U cells was significantly decreased (*P* = 0.0008) (**Figure 3D**), which was similar to MCF-7-shCENP-U and MCF-7-shControl cells (*P* = 0.0093) (**Figure S1D**). These data showed that knockdown CENP-U could repress the proliferation and colony formation of breast cancer cell lines.

To explore the underlying mechanism of CENP-U regulating the oncogenesis of breast cancer, cell cycle and apoptosis analysis were performed in the breast cancer cell line MDA-MB-231 by flow cytometry. Compared with the control group (**Figures 3E, F**), the percentage of cells in G2/M phase and apoptotic cells (indicated by increased percentage of Q2 and Q4 quadrants) was significantly increased in 231-shCENP-U cells. Meanwhile, the percentage of G1 phase markedly reduced, indicating an impaired capacity of DNA synthesis for the following cell division. Along with the result of the cell growth curve, we considered that silencing CENP-U could arrest the cell at the G2/M phases and induce breast cancer cell apoptosis.

### Up-Regulation of CENP-U Promotes the Proliferative Ability of Breast Cancer Cells and Breast Normal Epithelial Cells

**Figure 4:**
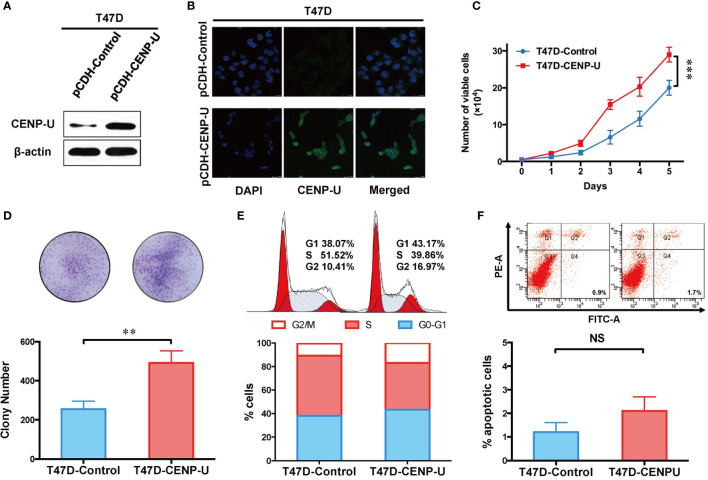
Up-regulation of CENP-U increased the proliferation of breast cancer cells. **(A)** The up-regulation of CENP-U expression was confirmed by western blotting in the subclones of T47D. **(B)** The over-expression of CENP-U did not change the location of CENP-U protein by immunofluorescence. **(C)** The cell growth curve of T47D-control cells and T47D-CENP-U cells. ***P < 0.001. **(D)** The colony number of T47D-control cells and T47D-CENP-U cells. **(E)** Cell cycle of T47D-control cells and T47D-CENP-U cells analysis were detected by flow cytometry. **(F)** Apoptosis analysis of T47D-control cells and T47D-CENP-U cells were performed by flow cytometry. The statistical analysis was performed with Student’s *t*-test. NS, not significant; **P < 0.01.

To confirm this observation in shCENP-U cells, we chose the low CENP-U expression cell lines (T47D and MCF-10A) to establish stable clones over-expressing CENP-U (T47D-CENP-U and MCF-10A-CENP-U) and control clones (T47D-Control and MCF-10A-Control), according to the expression of CENP-U in a series of breast cells. Over-expression of CENP-U was confirmed by western blot in the subclones of T47D (**Figure 4A**) and MCF-10A (**Figure S2A**). Immunofluorescence analysis confirmed that over-expression of CENP-U did not change the location of CENP-U protein (**Figure 4B** and **Figure S2B**). The proliferation rate of CENP-U over-expressing groups was significantly higher compared to that of control cells both in T47D (**Figure 4C**) and MCF-10A (**Figure S2C**) (*P* < 0.001) during the 5 days’ culture. The colony forming ability was apparently enhanced in T47D-CENP-U cells compared with T47D-Control cells (*P* = 0.0053) (**Figure 4D**), while the difference in colony numbers between MCF-10A-CENP-U cells and MCF-10A-Control cells was not significant (*P* = 0.0639) (**Figure S2D**), which might be related to the limited colony formation ability of MCF-10A. Therefore, we conducted a soft agar colony experiment to explore the role of CENP-U in colony formation in MCF-10A. For 5 weeks of culture, compared with the control group, the number of soft agar colonies was significantly increased (*P* = 0.0210) (**Figure S2E**). Up-regulation of CENP-U led to an increase in G1 and G2/M phase (**Figure 4E**), indicating that CENP-U could promote breast cancer cell mitosis. However, a similar cells number of apoptosis was observed between T47D-CENP-U and T47D-Control cells (*P* = 0.0967) (**Figure 4F**). These results demonstrated that up-regulation of CENP-U raised the proliferation of breast cancer cells by promoting cells into mitosis.

### CENP-U May Activate the PI3K/AKT/NF-κB Signaling Pathway During Breast Cancer Tumorigenesis

**Figure 5:**
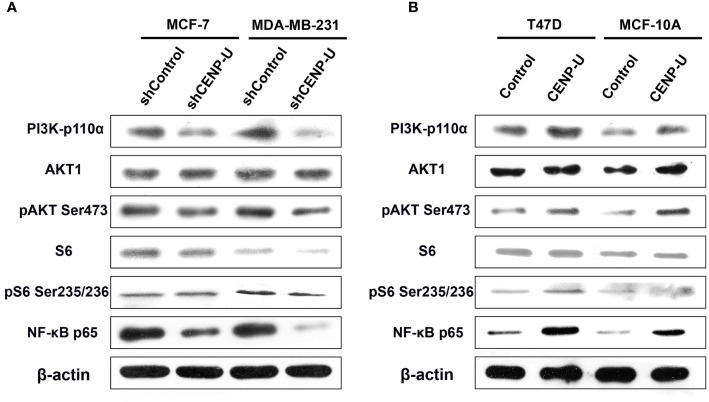
CENP-U may activate PI3K/AKT/NF-κB signaling pathway during breast cancer tumorigenesis. **(A)** PI3K/AKT/NF-κB pathway activity was inhibited in shCENP-U cells. **(B)** PI3K/AKT/NF-κB pathway was activated in cells with CENP-U over-expression compared to corresponding control cells; CENP-U also affected PI3K/AKT/NF-κB signaling pathway activity of normal mammary epithelial cells.

Our previous researches focused on the relationship between PI3K/AKT/NF-κB pathway and tumorigenesis of breast cancer cells (37). To better understand the underlying mechanism of CENP-U during oncogenesis of breast cancer, we compared activation of PI3K/AKT/NF-κB pathway in response to CENP-U over-expression and knockdown. Compared with the control cells, a significant decrease of PI3K-p110α subunit, S6 and NF-κB p65 subunit was observed in shCENP-U cancer cells, and phosphorylation sites protein of AKT Ser473 and S6 Ser235/236 also decreased significantly (**Figure 5A**). On the other hand, PI3K/AKT/NF-κB pathway was activated in cells with CENP-U over-expression compared to corresponding control cells (**Figure 5B**-Line1). In addition to breast cancer cell lines, CENP-U also affected PI3K/AKT/NF-κB signaling pathway activity of normal mammary epithelial cells (**Figure 5B**-Line 2). These data indicated that CENP-U may act as an oncogene in breast cancer by regulating the activity of the PI3K/AKT/NF-κB pathway.

### CENP-U Regulates the Tumor Growth of Breast Cancer Cells In Vivo

**Figure 6:**
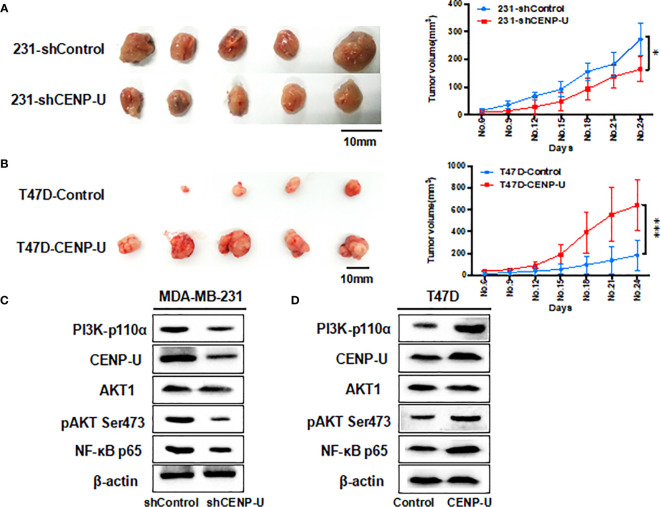
CENP-U regulates the tumor growth of breast cancer cells *in vivo.* **(A)** The xenograft tumors from the 231-shCENP-U cells were much smaller than those from the 231-shControl cells (*P* = 0.0329). **(B)** The xenograft tumors from the T47D-CENP-U cells were much bigger than those from the T47D-control cells. **(C)** PI3K/AKT/NF-κB pathway activity was inhibited in shCENP-U xenograft tumors compared to shControl xenograft tumors. **(D)** PI3K/AKT/NF-κB pathway was activated in xenograft tumors with CENP-U over-expression compared to control xenograft tumors (*P* < 0.0001). *P < 0.01, ***P < 0.001.

Xenograft tumor assays were performed through injecting 231-shCENP-U cells and 231-shControl cells into the mammary fat pads of female athymic nude mice (n = 5 per group). The *in vivo* experiment revealed that the xenograft tumors of 231- shControl cells were much larger than xenograft tumors of 231-shCENP-U cells (*P* = 0.0329) (**Figure 6A**). These results showed that depletion of CENP-U expression inhibited breast cancer cells proliferation *in vivo*, indicating that CENP-U positively regulated cancer tumorigenesis. In order to verify this result, we conducted the same experiments *in vivo* using T47D-CENP-U cells and T47D-control. Xenograft tumors from T47D-CENP-U cells were found to be much larger than tumors from T47D control cells (*P* < 0.0001) (**Figure 6B**). Compared with the shControl xenograft tumors, an obviously decrease of PI3K-p110α subunit and NF-κB p65 subunit was observed in shCENP-U xenograft tumors, and phosphorylation sites protein of AKT Ser473 also decreased significantly (**Figure 6C**). On the other hand, compared to control xenograft tumors, PI3K/AKT/NF-κB pathway was activated in xenograft tumors with over-expressed CENP-U (**Figure 6D**).

## Discussion

It has been demonstrated that the abnormal expression or activation of CENPs plays a significant part in cancer tumorigenesis and progression (38). In the current research, we verify the proto-oncogene effect of CENP-U, an important resident centromere-binding protein, on proliferation and tumorigenesis of breast cancer, suggesting CENP-U is a potential molecular target for anti-proliferation. Recent researches confirmed that CENP-U was aberrantly expressed in breast cancer analyzed by Gene Chip (19). This study is the first to demonstrate that CENP-U serves as a novel proto-oncogene that regulates protein levels in breast cancer tumorigenesis.

Our current data showed that the percentage of CENP-U positive cells in DICS and IDC tissues was higher than that of matched adjacent histological normal tissues. Intriguingly, a significantly increased CENP-U expression was found in IDC compared to DICS, which was consistent with previous results (22). CENP-U expression in cancer cells, compared with normal tissues, was elevated, and CENP-U positive cells in IBC were higher than DICS, indicating that CENP-U expression levels increased with the development of breast cancer. This is the first report on the location of CENP-U protein both in the nucleus and cytoplasm of four breast cancer cell lines and normal epithelial cell line. Early studies about the subcellular localization of CENP-U in normal tissue showed that CENP-U is mainly distributed in the nucleus (30–32), but it remains unclear in cancer cells. In malignant cells, CENP-A or CENP-C would appear non-chromatin ectopic expression (8, 37). Ectopic expression of this malignancy in CENPs may explain why the visible expression of CENP-U in the cytoplasm.

Our results confirmed that the ability of proliferation and colony formation of shCENP-U cells was significantly suppressed *in vitro* and *in vivo*. Otherwise, the amplified expression of CENP-U could not only facilitate the proliferation of breast cancer cells, which simultaneously increase normal breast epithelial cell proliferation. A study on the model of chicken DT40 cell line found that CENP-U gene knockout was not lethal, but could slow down cell proliferation (32), which was consistent with our study.

The ability of proliferation and colony formation of shCENP-U cells was significantly suppressed compared with shControl cells, while the portion of apoptotic cells and G2/M cells was higher than that in shCENP-U cells. PI3K-p110α, S6, NF-κB p65, AKT Ser473, and S6 Ser235/236 were lower in shCENP-U cells. Otherwise, the ability of proliferation and colony formation of pCDH-CENP-U cells was increased compared with the pCDH-Control cells. And over-expression CENP-U induced cells into mitosis (G2/M). PI3K-p110α and NF-κB p65 were up-regulated in pCDH-CENP-U cells. Also, the expression of AKT Ser473 and S6 Ser235/236 were detected higher in pCDH-CENP-U cells. Arimura et al. (37) reported that in CENP-U gene knockout cells when the cell cycle checkpoint (checkpoint) was not activated, the defective protein would prolong cell division period, which was coincident with our results. We have detected the proportion of apoptotic cells in stable pCDH-CENP-U and found: knockdown CENP-U led to apoptosis of breast cancer cells.

Our previous researches were focused on the relationship between PI3K/AKT/NF-κB pathway activity and tumorigenesis of breast cancer cells (39). Compared with shControl cells, PI3K-p110α, S6, NF-κB p65, AKT Ser473, and S6 Ser235/236 were lower in shCENP-U cells. Otherwise, PI3K-p110α and NF-κB p65 were up-regulated in pCDH-CENP-U cells and the expression of AKT Ser473 and S6 Ser235/236 were higher in pCDH-CENP-U cells. Kung et al. (40) indicated that CENP-E small molecule inhibitors can effectively repress breast cancer cells growth and induce apoptosis, and PI3K/AKT signaling pathway and apoptosis signaling pathway plays an important role in the anti-cancer drug treatment. This study demonstrated that CENP-U may accelerate breast cancer cells proliferation by activating PI3K/AKT/NF-κB signaling pathway. However, further study needs to be warranted to reveal the concrete mechanism of the gap between CENP-U up-regulation and the activation of PI3K/AKT pathway.

In summary, the CENP-U over-expression in breast cancer tissues, and the repression of CENP-U can induce early apoptosis by delayed mitotic withdrawal, and activate the PI3K/AKT/NF-κB signaling pathway to inhibit cancer cell proliferation. Therefore, CENP-U may act as a potential molecular target to treat breast cancer.

## Data Availability Statement

The original contributions presented in the study are included in the article/**Supplementary Material**. Further inquiries can be directed to the corresponding author.

## Ethics Statement

The studies involving human participants were reviewed and approved by the Institutional Review Board of Tianjin Medical University Cancer Institute and Hospital. The patients/participants provided their written informed consent to participate in this study. The animal study was reviewed and approved by the Institutional Review Board of Tianjin Medical University Cancer Institute and Hospital.

## Author Contributions

XH, YQ, and LC have contributed equally to this work. JZ is the corresponding author to this work. JZ designed this research topic. XH, YQ, and LC designed the research steps. XY, DZ, JL, SZ, and ZS collected data. XH, LC, and YQ analyzed data. YQ and JZ wrote the manuscript. All authors contributed to the article and approved the submitted version.

## Funding

This work was supported by the National Natural Science Foundation of China (grant number 81672623)

## Conflict of Interest

The authors declare that the research was conducted in the absence of any commercial or financial relationships that could be construed as a potential conflict of interest.

The handling Editor declared a shared affiliation, though no other collaboration, with the authors.

## Supplementary Material

Supplementary Figure 1 Depletion of CENP-U expression inhibits breast cancer cell proliferation in MCF-7. **(A)** CENP-U knockdown was confirmed by western blotting in the subclones of MCF-7. **(B)** Down-regulation of CENP-U expression did not change the location of CENP-U protein by immunofluorescence. **(C)** The cell growth curve of MCF-7-shControl cells and MCF-7-shCENP-U cells. **(D)** The colony number of MCF-7-shControl cells and MCF-7-shCENP-U cells.

Supplementary Figure 2 Up-regulation of CENP-U increased the proliferation of breast normal epithelial cells. **(A)** The up-regulation of CENP-U expression was confirmed by western blotting in the subclones of MCF-10A. **(B)** The over-expression of CENP-U did not change the location of CENP-U protein by immunofluorescence. **(C)** The cell growth curve of MCF-10A-Control cells and MCF-10A -CENP-U cells. **(D, E)** The colony number of MCF-10A-Control cells and MCF-10A -CENP-U cells.
