# Supplementary figures and images for: Over-Expression of Centromere Protein U Participates in the Malignant Neoplastic Progression of Breast Cancer
Source: Front Oncol. 2021 Mar 23;11:615427. doi: 10.3389/fonc.2021.615427 (PMC8021899; doi:10.3389/fonc.2021.615427)

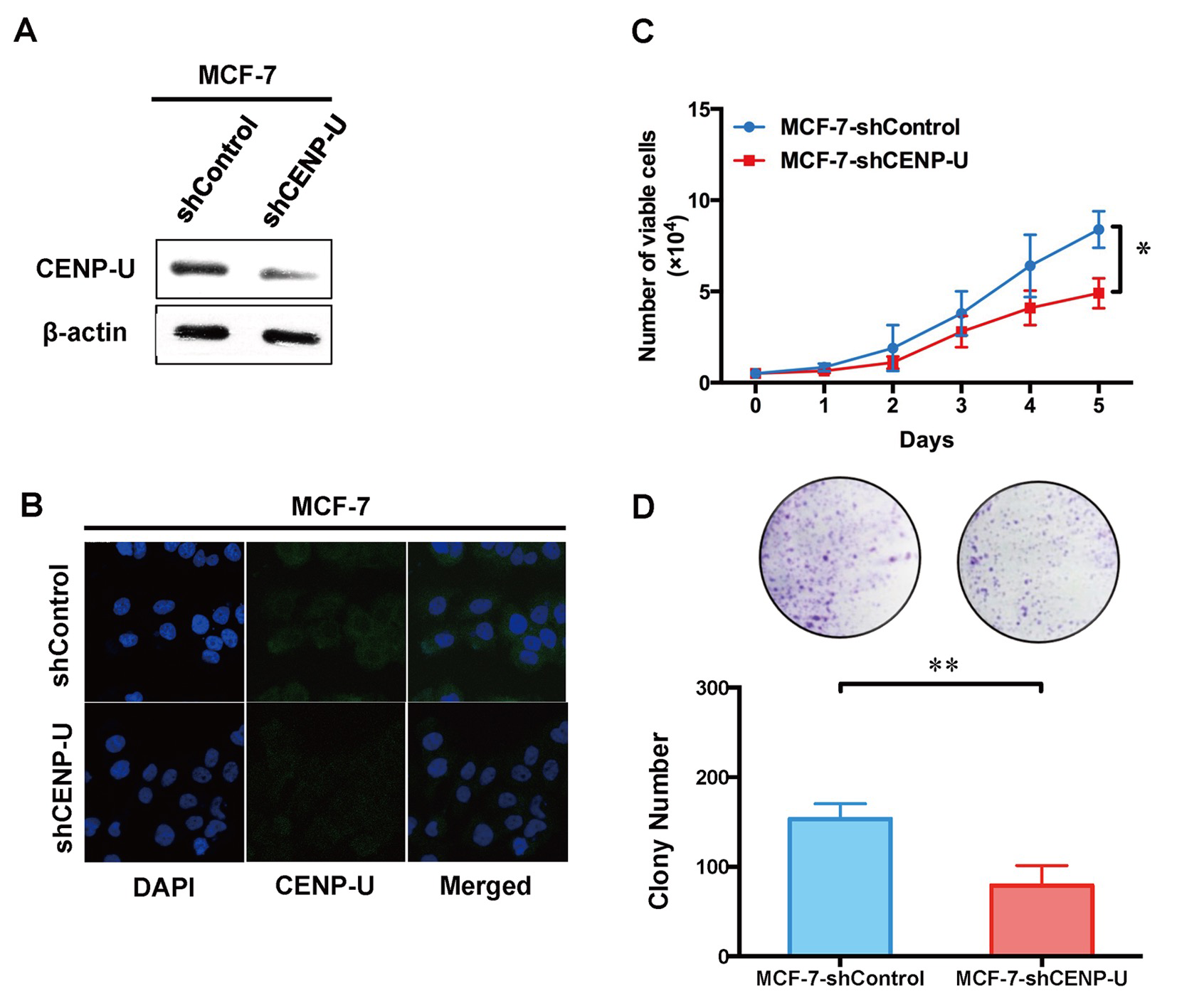

Supplement: Supplementary Figure 1 — Depletion of CENP-U expression inhibits breast cancer cell proliferation in MCF-7. (A) CENP-U knockdown was confirmed by western blotting in the subclones of MCF-7. (B) Down-regulation of CENP-U expression did not change the location of CENP-U protein by immunofluorescence. (C) The cell growth curve of MCF-7-shControl cells and MCF-7-shCENP-U cells. (D) The colony number of MCF-7-shControl cells and MCF-7-shCENP-U cells. [file Image_1.tif]

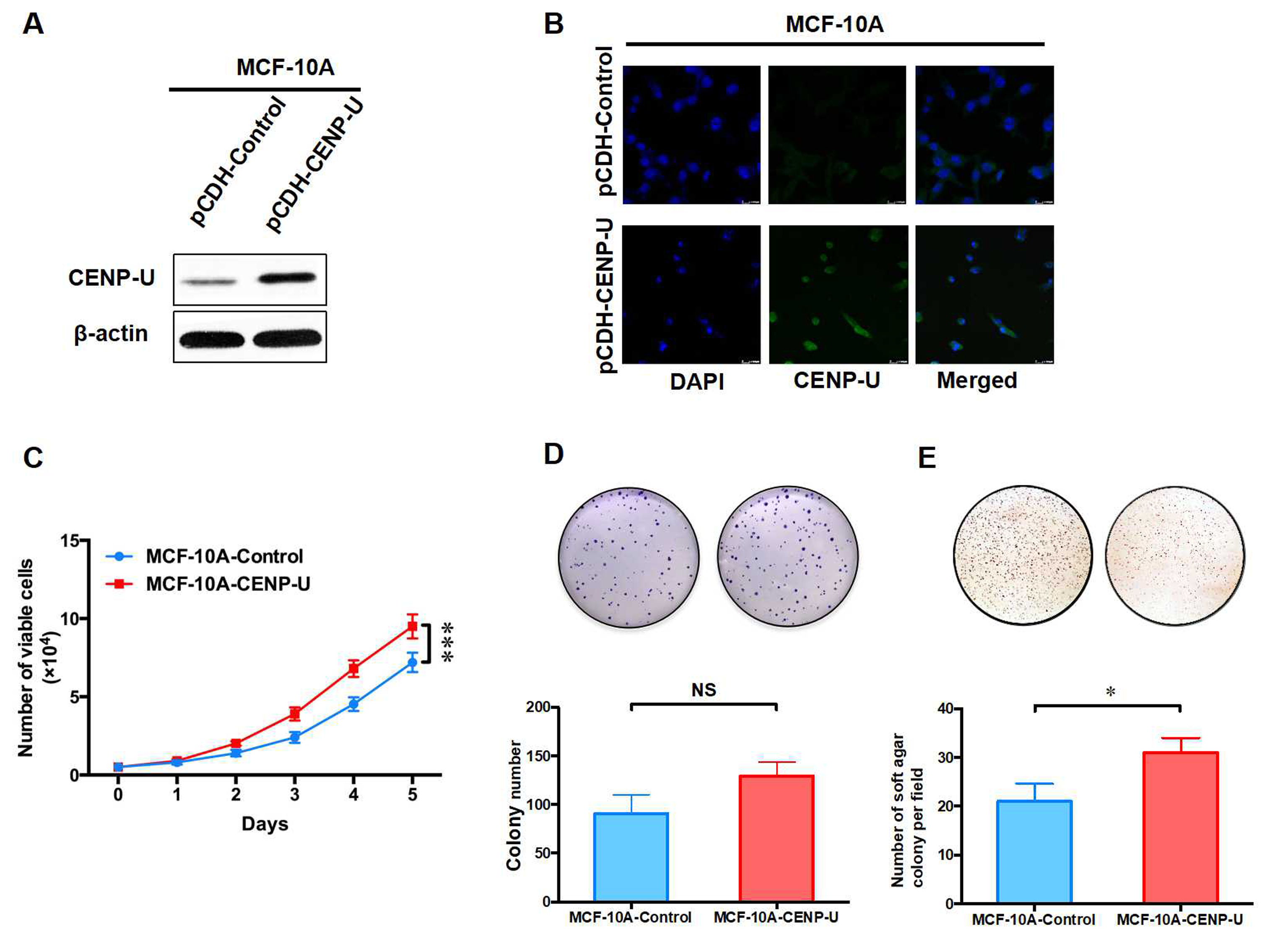

Supplement: Supplementary Figure 2 — Up-regulation of CENP-U increased the proliferation of breast normal epithelial cells. (A) The up-regulation of CENP-U expression was confirmed by western blotting in the subclones of MCF-10A. (B) The over-expression of CENP-U did not change the location of CENP-U protein by immunofluorescence. (C) The cell growth curve of MCF-10A-Control cells and MCF-10A -CENP-U cells. (D, E) The colony number of MCF-10A-Control cells and MCF-10A -CENP-U cells. [file Image_2.tif]
